# Supplementary material for: Distinct mesenchymal cell states mediate prostate cancer progression
Source: Nat Commun. 2024 Jan 8;15:363. doi: 10.1038/s41467-023-44210-1 (PMC10774315; doi:10.1038/s41467-023-44210-1)
Supplement: Supplementary file 1 — Supplementary Information [file 41467_2023_44210_MOESM1_ESM.pdf]

Supplementary Material

Distinct mesenchymal cell states mediate prostate cancer progression

Hubert Pakula[1,*], Mohamed Omar[1,2,*], Ryan Carelli[1,*], Filippo Pederzoli[1], Giuseppe Nicolò Fanelli[1,3], Tania Pannellini[1], Fabio Socciarelli[1], Lucie Van Emmenis[1], Silvia Rodrigues[1], Caroline Fidalgo-Ribeiro[1], Pier V. Nuzzo[1], Nicholas J. Brady[1], Wikum Dinalankara[1], Madhavi Jere [1], Itzel Valencia[1], Christopher Saladino[1], Jason Stone[1], Caitlin Unkenholz[1], Richard Garner[1], Mohammad K. Alexanderani[1], Francesca Khani[1], Francisca Nunes de Almeida[4], Cory Abate-Shen[4,5,6,7,8], Matthew B. Greenblatt[1], David S. Rickman[1], Christopher E. Barbieri[2,9], Brian D. Robinson[1,2,9], Luigi Marchionni[1], Massimo Loda[1,2,10,11]

Supplementary Figures

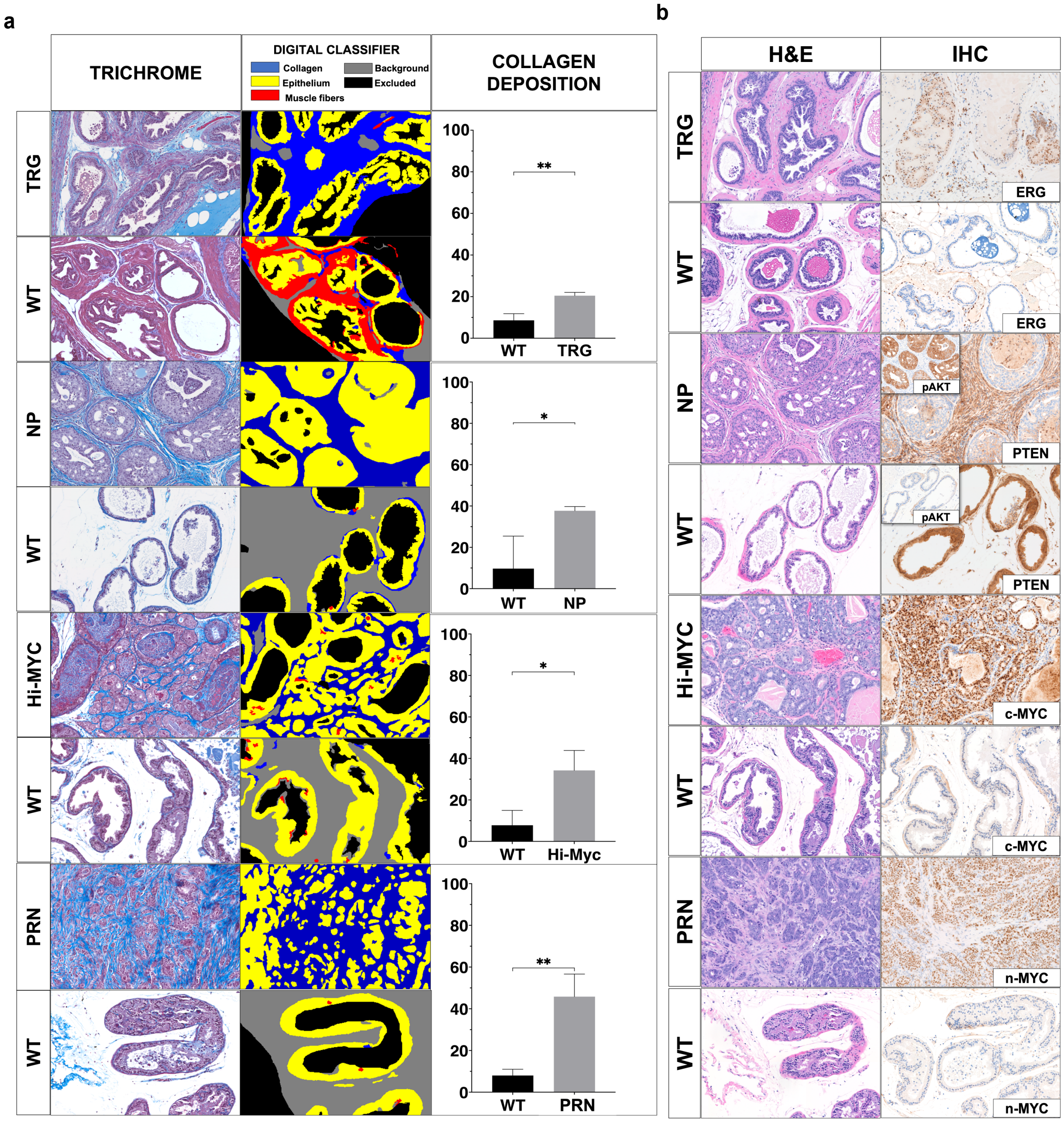

Supplementary Figure 1. GEMMs have increased stromal formation compared to their WT counterparts.

(a) Representative images of Masson's trichrome show the increasing collagen deposition in tumor models according to the aggressiveness of the disease (left panel). HALO-based digital classifier categorizes tissue into collagen, epithelium, muscle fiber, and background components (middle panel). Collagen deposition is significantly enriched in *NP*, *Hi-MYC,* and *PRN* models compared to their respective WTs (ns = not significant; * = $p<0.05$; ** = $p<0.01$; 2-tailed unpaired t-test; right panel). Bars in the histograms represent the mean values of the collagen deposition percentage. Error bars are the Standard Deviation SD. For each model, three different samples from GEMM and three corresponding WT mice have been analyzed. Magnification for all images is 200x. Scale bar: 300μm.

(b) Representative H&E and IHC images showing stromal reaction in the presence of characteristic GEMM proteins (*T-ERG* n=4, WT n=3; *NP*: n=3, WT: n=3; *Hi-MYC*: n=3, WT: n=3; *PRN*: n=3, WT: n=3, one representative image for each model). Magnification for all images 200x. Scalebar: 300μm.

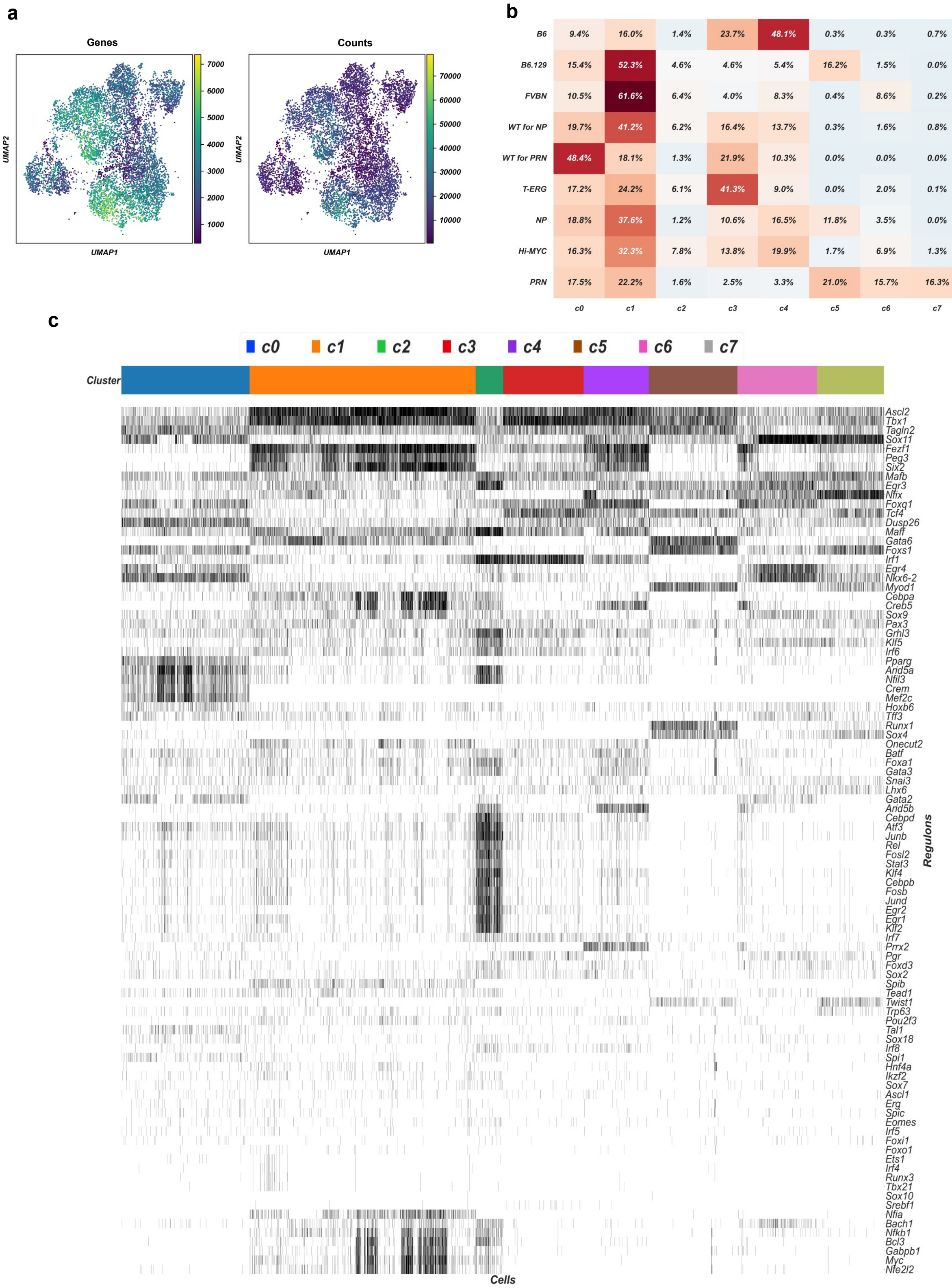
a
Genes
Counts
UMAP1
UMAP2
7000 6000 5000 4000 3000 2000 1000
70000 60000 50000 40000 30000 20000 10000
b
B6 9.4% 16.0% 1.4% 23.7% 48.1% 0.3% 0.3% 0.7%
B6.129 15.4% 52.3% 4.6% 4.6% 5.4% 16.2% 1.5% 0.0%
FVBN 10.5% 61.6% 6.4% 4.0% 8.3% 0.4% 8.6% 0.2%
WT for NP 19.7% 41.2% 6.2% 16.4% 13.7% 0.3% 1.6% 0.8%
WT for PRN 48.4% 18.1% 1.3% 21.9% 10.3% 0.0% 0.0% 0.0%
T-ERG 17.2% 24.2% 6.1% 41.3% 9.0% 0.0% 2.0% 0.1%
NP 18.8% 37.6% 1.2% 10.6% 16.5% 11.8% 3.5% 0.0%
Hi-MYC 16.3% 32.3% 7.8% 13.8% 19.9% 1.7% 6.9% 1.3%
PRN 17.5% 22.2% 1.6% 2.5% 3.3% 21.0% 15.7% 16.3%
c0 c1 c2 c3 c4 c5 c6 c7
c
c0 c1 c2 c3 c4 c5 c6 c7
Cluster
Ascl2 Tbx1 Tagln2 Sox11 Fezf1 Peg3 Six2 Mafb Egr3 Nfix Foxq1 Tcf4 Dusp26 Maff Gata6 Foxs1 Irf1 Egr4 Nkx6-2 Myod1 Cebpa Creb5 Sox9 Pax3 Grhl3 Klf5 Irf6 Pparg Arid5a Nfil3 Crem Mef2c Hoxb6 Tff3 Runx1 Sox4 Onecut2 Batf Foxa1 Gata3 Snai3 Lhx6 Gata2 Arid5b Cebpd Atf3 Junb Rel Fosl2 Stat3 Klf4 Cebpb Fosb Jund Egr2 Egr1 Klf2 Irf7 Prrx2 Pgr Foxd3 Sox2 Spib Tead1 Twist1 Trp63 Pou2f3 Tal1 Sox18 Irf8 Spi1 Hnf4a Ikzf2 Sox7 Ascl1 Erg Spic Eomes Irf5 Foxi1 Foxo1 Ets1 Irf4 Runx3 Tbx21 Sox10 Srebf1 Nfia Bach1 Nfkb1 Bcl3 Gabpb1 Myc Nfe2l2
Regulons
Cells

Supplementary Figure 2. Gene abundance, mouse models distribution, and regulons activity across different mesenchymal clusters.

(a) UMAP visualization of the eight mesenchymal clusters (n=8574 cells) colored by the number of detected genes (left) and Unique Molecular Identifiers (UMIs) (right).

(b) Heatmap showing the percentage of the different mesenchymal clusters in each mouse model. Three clusters (c0-c2) represent fibroblast states common to all genotypes, 5 clusters (c3-c7) are specific stromal responses to epithelial mutations. Stroma of two additional wildtype strains (*B6* and *B6.129*) varies in the different backgrounds.

(c) Heatmap of the binarized regulon activity in the different stromal clusters in the mouse scRNA-seq data. The activity of each regulon in each cell was computed using the AUCell algorithm within the SCENIC workflow and then binarized using automatic cutoffs into active (black) or non-active (white). Shown is heatmap of the binarized activity of significant regulons (right) across all mesenchymal cells grouped by their corresponding cluster (top).

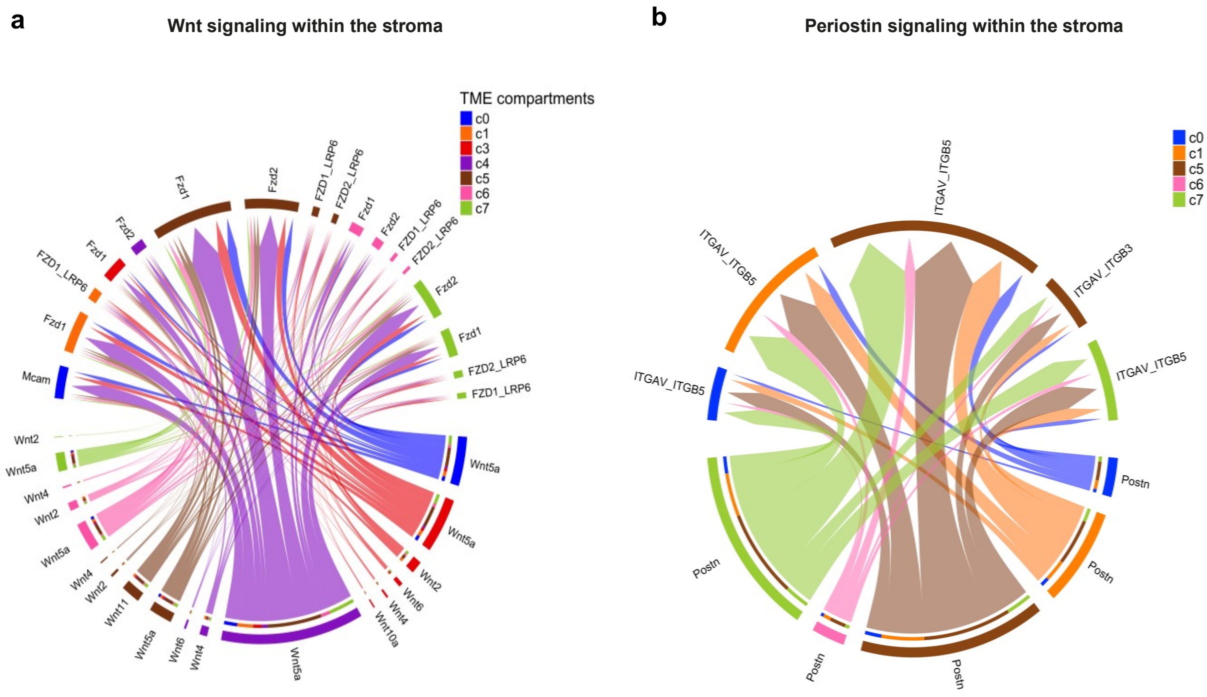

Supplementary Figure 3. Complex intercellular interactions within the PCa mesenchyme

(a) Significant stromal-stromal interactions mediated by the WNT signaling pathway.

(b) Significant stromal-stromal interactions mediated by the POSTN signaling pathway.

The size of the inner bars represents the signal strength received by their targets.

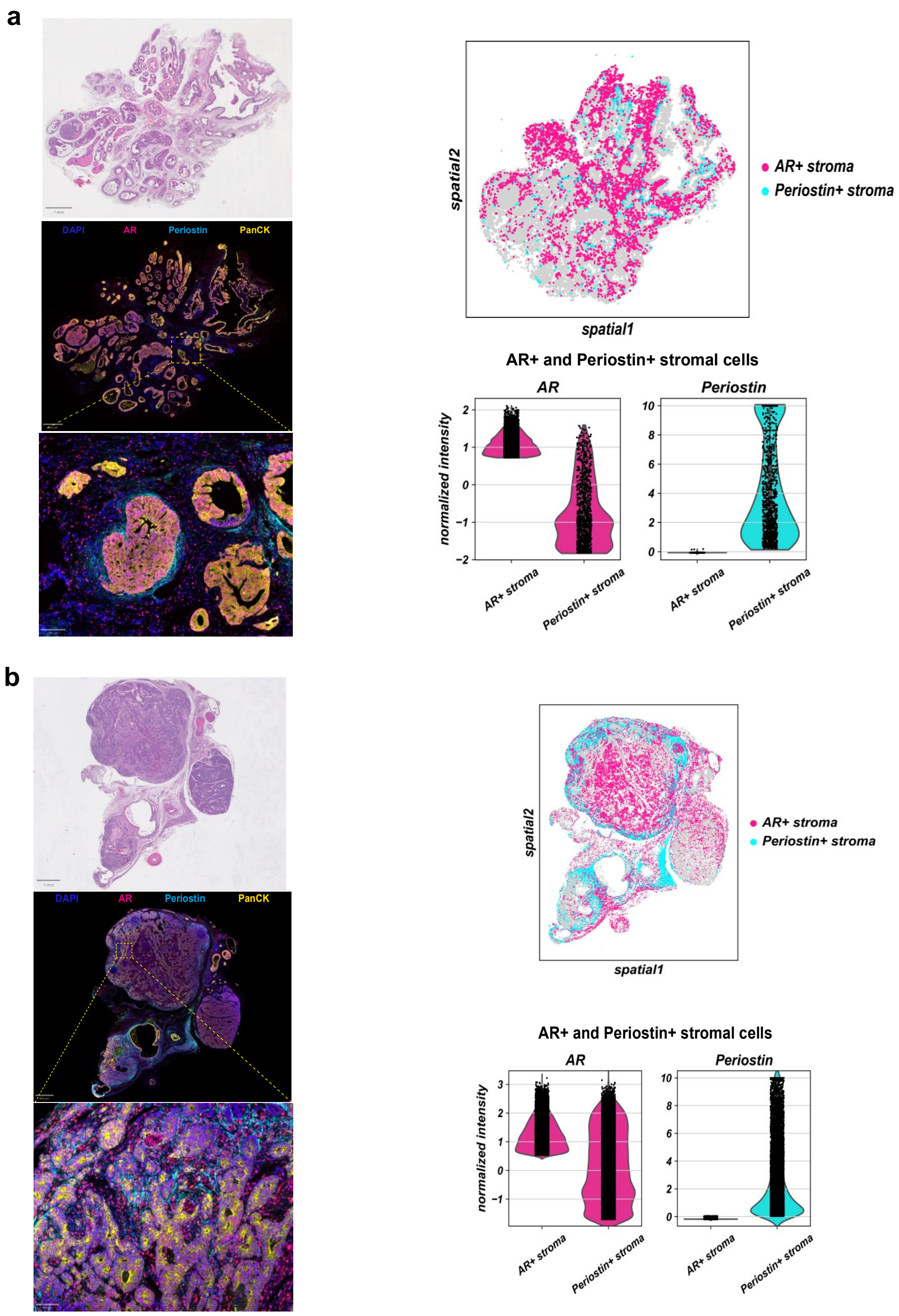

Supplementary Figure 4. Multispectral staining of prostate cancer tissue from mouse models of neuroendocrine prostate cancer (NEPC).

Multispectral immunohistochemistry (mIHC) of prostate cancer tissue from the DKO (n=1) (a)

and TKO (n=1) (b) mouse models of PCa with a panel of proteins including AR, Periostin, and PanCK. For each model (n=1), shown are the H&E-stained (top) and mIHC-stained (middle) tissue section (one for each model) (top), together with a high-resolution view of selected tissue region (one representative image for each model). The spatial plots on the right shows the overlay of the AR+ and Perisotin+ stromal cells on the original slides of DKO (n=68244 cells) and TKO (n=109074 cells). The violin plots compare the expression of AR and Periostin in the AR+ and Periostin+ stroma.

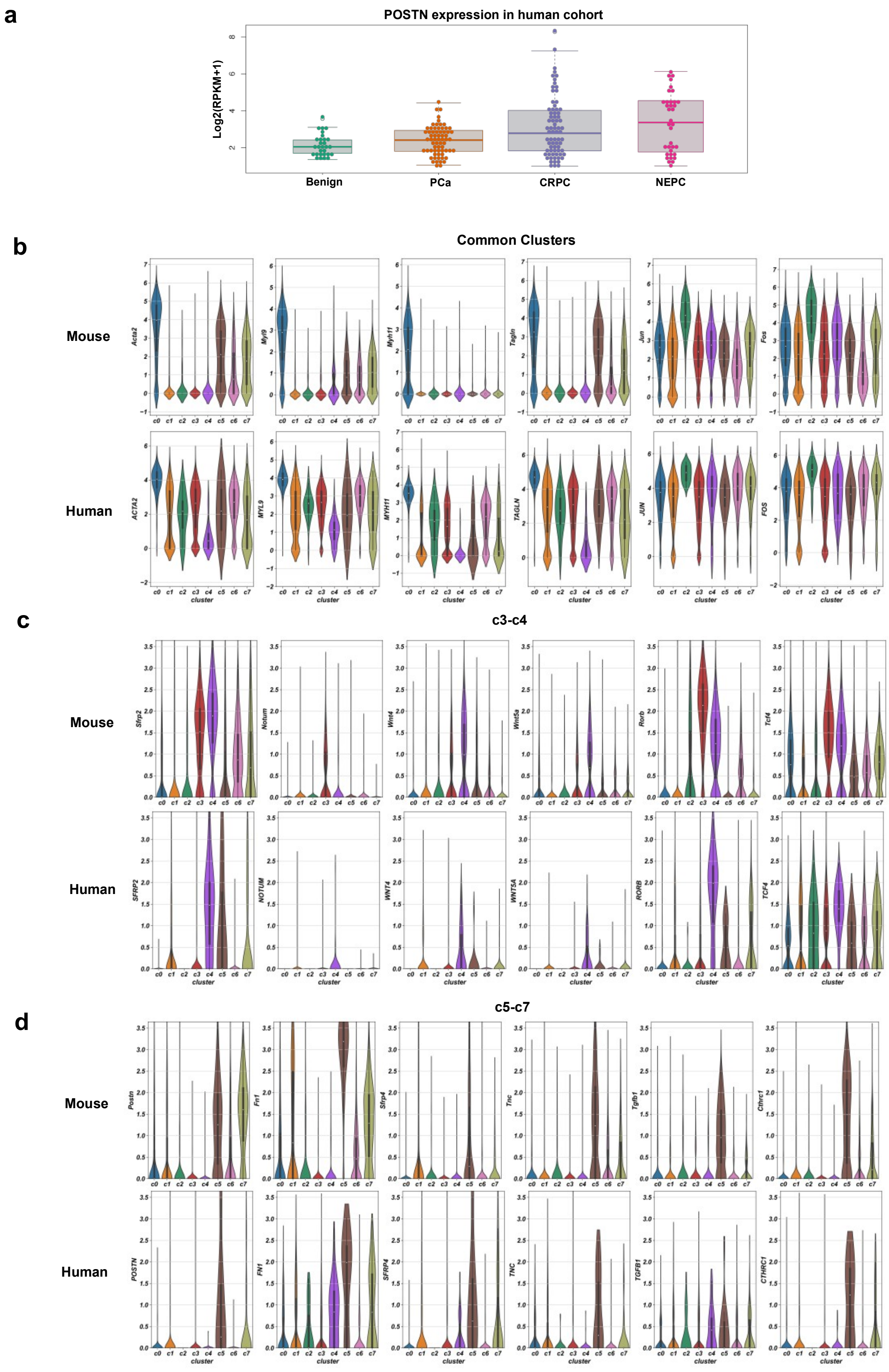
a
POSTN expression in human cohort
Log2(RPKM+1)
Benign
PCa
CRPC
NEPC
b
Common Clusters
Mouse
Human
Acta2
Myl9
Myh11
Tagln
Jun
Fos
ACTA2
MYL9
MYH11
TAGLN
JUN
FOS
cluster
c
c3-c4
Sfrp2
Notum
Wnt4
Wnt5a
Rorb
Tcf4
SFRP2
NOTUM
WNT4
WNT5A
RORB
TCF4
d
c5-c7
Postn
Fn1
Sfrp4
Tnc
Tgfb1
Cthrc1
POSTN
FN1
SFRP4
TNC
TGFB1
CTHRC1

Supplementary Figure 5. The transcriptional similarities between the stromal clusters identified in mouse models and those in human data.

(a) Boxplots of *POSTN* expression in WCM clinical cohort 29 benign prostate, 66 prostate adenocarcinoma (PCa), 73 castrate resistant prostate adenocarcinoma (CRPC) and 36 neuroendocrine prostate cancer (NEPC) clinical samples. (p-value < 0.01, Wilcoxon test, RPKM: reads per kilobase million).

(b-d) Violin plots for the expression of marker genes for the common clusters c0-c2 (b), the WNT-associated clusters c3-c4 (c), and the PRN-associated clusters c5-c7 (d). The width of the violins at different values represents the density of the data. The embedded box plots display the median of the data (white dot), the bounds of the box represent the 25th and 75th percentiles (interquartile range), and the data within these bounds represent the minima and maxima of the non-outlying data.

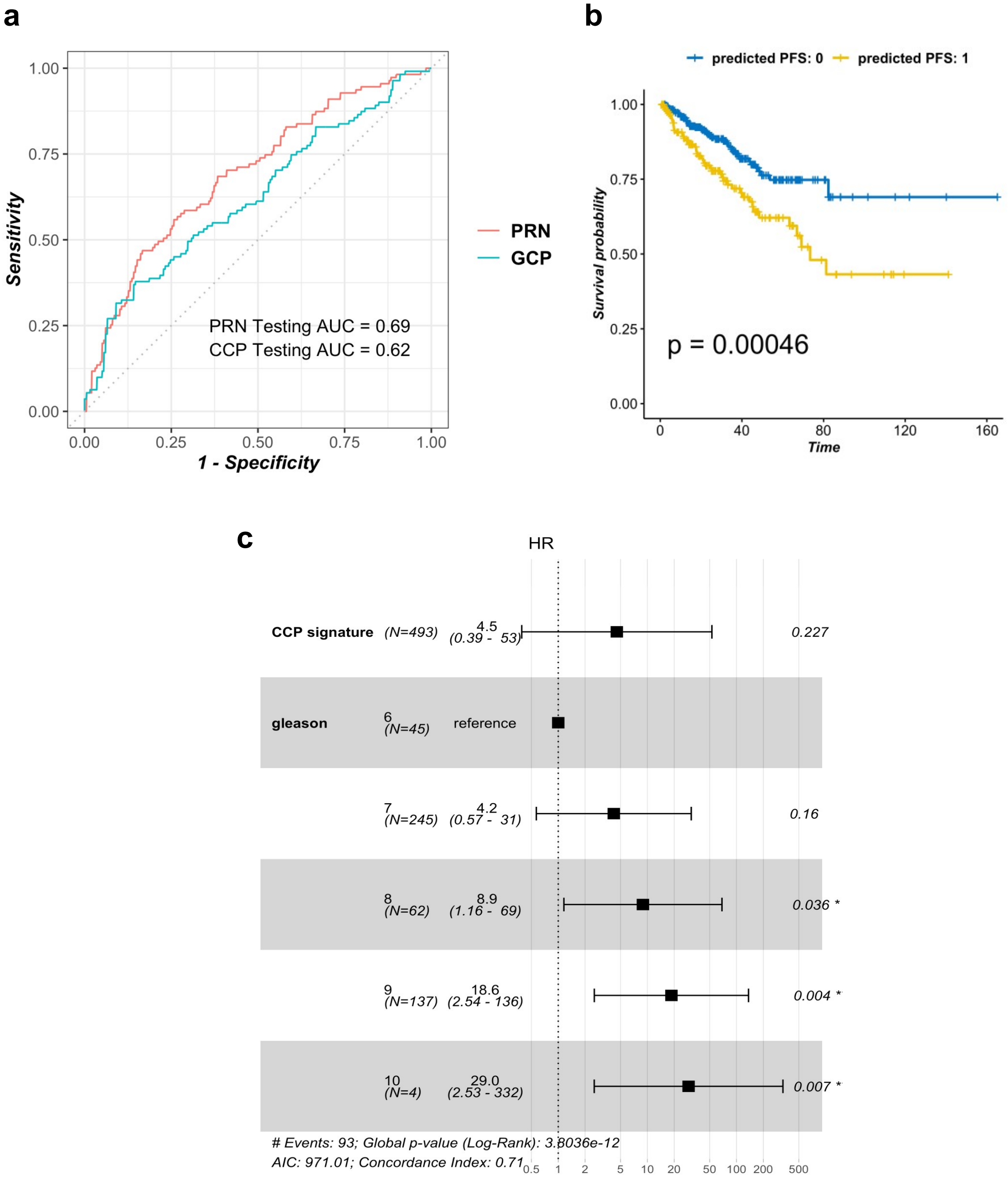

Supplementary Figure 6. The predictive and prognostic performance of the cell-cycle progression signature in human data.

(a) Receiver Operating Characteristics (ROC) curves comparing the Area Under the Curve (AUC) of the cell-cycle progression (CCP) signature (blue) and the PRN stromal signature (red) in the testing cohort (n=309).

(b) Kaplan-Meier (KM) survival plots showing the association between the CCP signature and progression-free survival in the TCGA cohort (n=439). P: p-value using the log-rank test.

(c) Forest plot showing the hazard ratios (HR) (central black square) and 95% confidence interval (CI) (horizontal lines) of the CCP signature and progression-free survival in the TCGA cohort (n=439) after adjusting for Gleason grade in a multivariate Cox proportional hazards model. Significance, indicated by an asterisk, is based on the p-value from the Wald test in the Cox model (*: p-value <0.05).

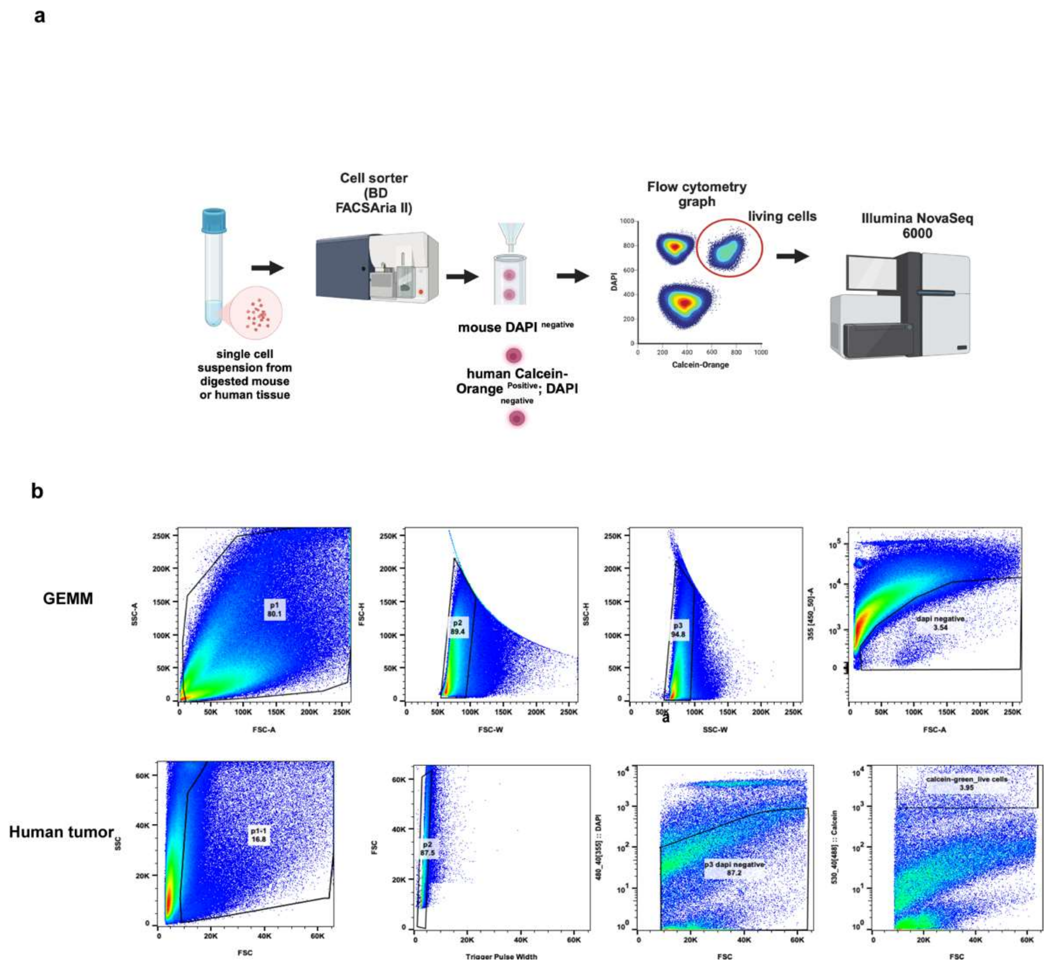

Supplementary Figure 7: Gating strategy for isolation of living cells from enzymatically digested mouse and human tissues for 10x Chromium profiling

(a) Schematic representation of the procedure for isolating mouse and human prostate cancer living cells for 10x Chromium profiling using fluorescence-activated cell sorting (FACS). Created with BioRender.com.

(b) Gating strategy employed to isolate living cells from mouse and human prostate tissues for 10x Chromium profiling. Representative FACS plots are featuring genetically engineered mouse models (GEMM), as well as human prostate cancer tissue, illustrating the approach applied in disease states.
