# Supplementary material for: Distinct mesenchymal cell states mediate prostate cancer progression
Source: Nat Commun. 2024 Jan 8;15:363. doi: 10.1038/s41467-023-44210-1 (PMC10774315; doi:10.1038/s41467-023-44210-1)
Supplement: Supplementary file 2 — Description of Additional Supplementary Files [file 41467_2023_44210_MOESM2_ESM.pdf]

Description of Additional Supplementary Files

Supplementary Data 1. Genetically engineered mouse models and corresponding wild types used in the present study.

Number of mice sequenced for each model and wildtype used in the present study, together with associated numbers of sequenced cells and transcripts.

Supplementary Data 2. Canonical gene markers used for cell type identification.

List of canonical lineage gene markers used for cell type identification.

Supplementary Data 3. Significant ligand-receptor interactions in the mouse scRNA-seq data.

The ligand-receptor (L-R) interactions were computed between the epithelium, stroma, and immune compartments, and also between different cell types in the three compartments. L-R interactions were compared between genetically-engineered mouse models (GEMMs) and wildtypes (WT). The table includes all the significant up-regulated interactions (p-value<0.05) in all GEMMs compared to WTs (sheet 1*), T-ERG* compared *to Hi-MYC* (sheet 2), *Hi-MYC* compared to *T-ERG* (sheet 3), *NP* compared to *Hi-MYC* (sheet 4), *T-ERG* compared to NP (sheet 5), and *PRN* compared to its WT (sheet 6). Up-regulated interactions were identified based on differential gene expression analysis using a two-sided Wilcoxon rank-sum test. P-values for communication probabilities are computed from a permutation test by randomly permuting the cell group labels (100 permutations), and then recalculating the communication probability.

Supplementary Data 4. Differentially expressed genes (DEGs) for each stromal cluster compared to the remaining clusters in the mouse scRNA-seq data.

DEGs were identified by comparing each cluster to the remaining clusters using the MAST approach employing a hurdle model tailored for scRNA-seq data. The p-values have been adjusted for multiple comparisons using the Bonferroni method.

Supplementary Data 5. The PRN signature for predicting prostate cancer metastasis.

The signature consists of 13 gene pairs with each including a gene up- and another down-regulated in the *PRN* mesenchyme (c5-c7). Pairs vote for metastasis if the 1st gene is overexpressed relative to second. A patient with >=7 votes will be predicted to have metastasis.

Supplementary Data 6. Human PCa samples.

List of human PCa samples used in the present study for scRNA-seq.

Supplementary Data 7. Multiplex immunohistochemistry (mIF) antibody panels.
