# Supplementary material for: Distinct mesenchymal cell states mediate prostate cancer progression
Source: Nat Commun. 2024 Jan 8;15:363. doi: 10.1038/s41467-023-44210-1 (PMC10774315; doi:10.1038/s41467-023-44210-1)
Supplement: Supplementary file 10 — Reporting Summary [file 41467_2023_44210_MOESM10_ESM.pdf]

nature portfolio

Corresponding author(s): Massimo Loda

Last updated by author(s): Nov 3, 2023

Reporting Summary

Nature Portfolio wishes to improve the reproducibility of the work that we publish. This form provides structure for consistency and transparency in reporting. For further information on Nature Portfolio policies, see our Editorial Policies and the Editorial Policy Checklist.

Statistics

For all statistical analyses, confirm that the following items are present in the figure legend, table legend, main text, or Methods section.

n/a	Confirmed	
☐	☒	The exact sample size (*n*) for each experimental group/condition, given as a discrete number and unit of measurement
☒	☐	A statement on whether measurements were taken from distinct samples or whether the same sample was measured repeatedly
☐	☒	The statistical test(s) used AND whether they are one- or two-sided<br>*Only common tests should be described solely by name; describe more complex techniques in the Methods section.*
☐	☒	A description of all covariates tested
☐	☒	A description of any assumptions or corrections, such as tests of normality and adjustment for multiple comparisons
☐	☒	A full description of the statistical parameters including central tendency (e.g. means) or other basic estimates (e.g. regression coefficient) AND variation (e.g. standard deviation) or associated estimates of uncertainty (e.g. confidence intervals)
☐	☒	For null hypothesis testing, the test statistic (e.g. *F*, *t*, *r*) with confidence intervals, effect sizes, degrees of freedom and *P* value noted<br>*Give P values as exact values whenever suitable.*
☒	☐	For Bayesian analysis, information on the choice of priors and Markov chain Monte Carlo settings
☒	☐	For hierarchical and complex designs, identification of the appropriate level for tests and full reporting of outcomes
☒	☐	Estimates of effect sizes (e.g. Cohen's *d*, Pearson's *r*), indicating how they were calculated

Our web collection on statistics for biologists contains articles on many of the points above.

Software and code

Policy information about availability of computer code

Data collection	Total RNA libraries were sequenced on an Illumina NovaSeq 6000 instrument. The STAR aligner within CellRanger (10x Genomics, version 5.0.0) was used for the alignment of the single cell RNA seq reads and the generation of feature-barcode matrices.<br>The normalized bulk expression profiles used to train and validate the PRN and CCP signatures were retrieved from the Gene Expression Omnibus using the R package 'GEOquery' v2.58.0.<br>Whole slide images of the mouse and human sections stained with hematoxylin and eosin (H&E), trichrome, and multiplex immunohistochemistry (mIHC) were acquired using the Vectra Polaris Automated Quantitative Pathology Imaging System (Akoya Biosciences, Hopkinton, MA).<br>For the Visium data, H&E-stained images were acquired using the EVOS M7000 Automated Imaging System (10x objective, 3.45µm/pixel - Thermo Fisher Scientific, CA). Sequencing libraries were demultiplexed with bcl2fastq (Illumina). Spatial transcriptomics libraries were processed and aligned using the Space Ranger software (10x Genomics, version 2.0.1).
Data analysis	The custom scripts used for the analysis of the scRNA-seq and Visium spatial transcriptomics data are publicly available on GitHub at the following repository: https://github.com/MohamedOmar2020/pca_TME.git..<br>The quantification of collagen deposition in images stained with Masson's trichome staining was performed using the HALO software (Indica Labs, v3.3.2541, Albuquerque, US). The preprocessing and quantification of mIHC images were performed using the PathML library (v2.0.0).<br>The R programming language (v4.0.3) was used for performing the ligand-receptor interactions analyses using the CellChat tool (v1.1.3) and the survival analyses using the survival (v3.3.1) and survminer (v0.4.9) libraries. The gene regulatory network analysis was performed using the PySCENIC python library (v0.10.3).

For manuscripts utilizing custom algorithms or software that are central to the research but not yet described in published literature, software must be made available to editors and reviewers. We strongly encourage code deposition in a community repository (e.g. GitHub). See the Nature Portfolio guidelines for submitting code & software for further information.

Data

Policy information about availability of data

All manuscripts must include a data availability statement. This statement should provide the following information, where applicable:

- Accession codes, unique identifiers, or web links for publicly available datasets
- A description of any restrictions on data availability
- For clinical datasets or third party data, please ensure that the statement adheres to our policy

The single-cell RNA-seq data generated in this study has been deposited in the Gene Expression Omnibus (GEO) under the accession codes: GSE244267 and GSE244269. The processed count matrices for the single-cell RNA-seq, Visium spatial transcriptomics data, together with the expression matrix and phenotype labels of the natural history cohort have been deposited on Zenodo (https://zenodo.org/doi/10.5281/zenodo.7452769). The microscopy data reported in this paper will be shared by the lead contact. The remaining data are available within the Article, Supplementary Information or Source Data file.

Research involving human participants, their data, or biological material

Policy information about studies with human participants or human data. See also policy information about sex, gender (identity/presentation), and sexual orientation and race, ethnicity and racism.

Reporting on sex and gender

This study uses data from mouse models as well as patients with prostate cancer. All the human research subjects were self-identified as male.

Reporting on race, ethnicity, or other socially relevant groupings

While the data in Table S6 include ethnicity information, we did not gather data pertaining to socially relevant categorization variables.

Population characteristics

Human prostate tissue specimens (de-personalized) were obtained from patients undergoing radical prostatectomy at Weill Cornell Medicine under Institutional Review Board approval with informed consent (WCM IRB #1008011210, #1302013582).

Age distribution of patients:
41-50 (n=2); 51-60 (n=4); 61-70 (n=2); 71-80 (n=3); 81-90 (n=1); 91-100 (n=1)

The study included a total of 13 subjects, all of whom were males. No blinding, randomization, or exclusion criteria were applied. Of these, 9 samples (comprising 3 ERG-negative and 6 ERG-positive cases) were utilized for single-cell RNA sequencing studies, while 4 samples were employed for the mIHC Vectra Polaris staining.

Human samples (n= 6 ERG positive and n=3 ERG negative) are detailed in Table S6 which includes the following information:

Sample ID, Ethnicity, Pre-Prostatectomy PSA (ng/mL), Grade Group (Gleason score) of Dominant Nodule, Neoplastic cellularity, Pathologic Stage, Oncomine Findings:

- ERG+_1, 10.1, Grade Group 1 (Gleason score 3+3=6), 30%, pT2 pN0, TMPRSS2 - ERG fusion, CCNE1 c.617A>G, p.Tyr206Cys missense

-ERG+_2, 5.3, Grade Group 2 (Gleason score 3+4=7), 80%, pT3a pN0, TMPRSS2 - ERG fusion

- ERG+_3, 5.1 Grade Group 1 (Gleason score 3+3=6), 70%, pT2 pN0, TMPRSS2 - ERG fusion

- ERG+_4, 7.1 Grade Group 3 (Gleason score 4+3=7), NA, pT3a pN0, TMPRSS2 - ERG fusion; CDKN2A c.434T>C, p.Ile145Thr; BAP1 c.544G>C, p.Glu182Gln

- ERG+_5, , 3.2 Grade Group 3 (Gleason score 4+3=7), 60% ,pT2 pN0, TMPRSS2 - ERG fusion

- ERG+_6, , 13.4, Grade Group 2 (Gleason score 3+4=7), 50%, pT2 pN0 , TMPRSS2 - ERG fusion

- ERG-_1, 12.3, Grade Group 2 (Gleason score 3+4=7), 70%, pT3a pN0, ATP11B c.2563G>A, p.Ala855Thr- missense
EGFR c.3632G>C, p.Ter1211Serext*6 -stoploss

- ERG-_2, , 7.1 Grade Group 2 (Gleason score 3+4=7), 50% ,pT2 pN0, IDH1 c.394C>G, p.Arg132Gly- missense
EGFR c.3632G>C, p.Ter1211Serext*6 -stoploss

- ERG-_3, , 6.5 Grade Group 2 (Gleason score 3+4=7) with tertiary Gleason pattern 5), 50% ,NA ,ERG (negative), no gene mutation found

In addition, for mIHC Vectra Polaris we used 4 human biopsies described detailed in Table S6 which includes the following information: Patient ID, Ethnicity, Pre-Prostatectomy PSA (ng/mL), Grade Group (Gleason score) of Dominant Nodule, Neoplastic cellularity, Pathologic Stage and Additional notes

-PCa_1, 5,25 Grade Group 2 - Gleason Score: 3+4=7 30% pT2 pN0 NA
-PCa_2, 43.7 Grade group 5 - Gleason score: 4+5=9 10% pT3aN0 NA
-PCa_3, 24 "biopsy" Grade Group 2 (Gleason score 3+4=7) 65% NA Large cell neuroendocrine carcinoma and Prostatic adenocarcinoma, .
-PCa_4, 0.56 "Chips" NA 35% NA High grade carcinoma with neuroendocrine features and prostatic adenocarcinoma with thermal artifacts

All patients were male and tumors were classified based on histomorphology using a published pathologic classification system [Epstein et al., Am. J. Surg. Pathol. 38, 756-767 (2014)]. No other covariates were assessed.

Participants did not receive compensation for their participation in this project

Recruitment	For the human scRNA-seq data, we retrospectively selected patients who had voluntarily consented to provide samples for future research purposes.<br>The bulk expression profiles used for predictive modeling of metastasis were publicly available and were retrieved from the Gene Expression Omnibus.
Ethics oversight	Human prostate tissue specimens were obtained from patients undergoing radical prostatectomy at Weill Cornell Medicine under Institutional Review Board approval with informed consent (WCM IRB #1008011210, #1302013582).

Note that full information on the approval of the study protocol must also be provided in the manuscript.

Field-specific reporting

Please select the one below that is the best fit for your research. If you are not sure, read the appropriate sections before making your selection.

- [x] Life sciences
- [ ] Behavioural & social sciences
- [ ] Ecological, evolutionary & environmental sciences

For a reference copy of the document with all sections, see nature.com/documents/nr-reporting-summary-flat.pdf

Life sciences study design

All studies must disclose on these points even when the disclosure is negative.

Sample size	No statistical method was used to predetermine sample size. For the analyses employing single cell RNA-seq and spatial transcriptomics, no statistical methods were used to predetermine the sample size. For the predictive modeling of metastasis using bulk expression profiles, we included all publicly available primary tumor expression profiles from patients with prostate cancer (n=1239).
Data exclusions	No data was excluded
Replication	The code used to retrieve and process the expression profiles is publicly available to ensure complete reproducibility. This code can be accessed using the following link: https://github.com/MohamedOmar2020/pca_TME
Randomization	For the predictive modeling of metastasis, 75% of all samples (n=1239) were assigned to the training set (n=930) and 25% for the testing set (n=309). This training-testing ratio is a conventionally accepted ratio in predictive modeling since it ensures allocating most samples for training the model and preserving sufficient number of samples for testing the model's performance.<br>We used stratified sampling for allocating the samples to training or testing sets to ensure equal representation of important covariates (tumor stage, Gleason grade, PSA levels, and patient age) in both sets. These covariates were used due to their importance for the predicted label (prostate cancer metastasis).<br>For the single cell RNA-seq and spatial transcriptomics experiments, no randomization was performed as the focus was to investigate the composition of the tumor microenvironment in different stages of disease progression.
Blinding	Blinding was not relevant to our study since we do not compare patient groups.

Reporting for specific materials, systems and methods

We require information from authors about some types of materials, experimental systems and methods used in many studies. Here, indicate whether each material, system or method listed is relevant to your study. If you are not sure if a list item applies to your research, read the appropriate section before selecting a response.

Materials & experimental systems

n/a	Involved in the study
☐	☒ Antibodies
☐	☒ Eukaryotic cell lines
☒	☐ Palaeontology and archaeology
☐	☒ Animals and other organisms
☒	☐ Clinical data
☒	☐ Dual use research of concern
☒	☐ Plants

Methods

n/a	Involved in the study
☒	☐ ChIP-seq
☐	☒ Flow cytometry
☒	☐ MRI-based neuroimaging

Antibodies

Antibodies used

Antibodies are detailed in Supplementary Table 7, which includes the following information:
Target, AbID, Primary ab Name, clone, Clonality, Lot, Host species, Primary ab dilution, Detection Fluorophore, Fuorophore Dilution RRID:
-Gpx3, ab256470, Anti-Glutathione Peroxidase 3/GPx-3 antibody [EPR22815-112], EPR22815-112, Monoclonal,GR3284787-2, Rabbit, 1/200, Opal 480, 1/75, N/A
-C3, ab200999, Anti-C3 antibody [EPR19394], EPR19394, Monoclonal, GR3336457-1, Rabbit, 1/10 000, Opal 520, 1/75, RRID:AB_2924273
- Wif1, ab155101, Recombinant Anti-WIF1 antibody [EPR9385], EPR9385, Monoclonal, n/a, Rabbit 1/5000 Opal 520 1/75
-Synaptophysin BD Biosciences (611880) Purified Mouse Anti-Synaptophysin 2/synaptophysin (RUO), Monoclonal,9352253, Mouse 1/100 Opal 520 1/75, RRID:AB_399360
-Periostin ab215199 Recombinant Anti-Periostin antibody [EPR20806], EPR20806, Monoclonal, GR3237082-6, Rabbit, 1/1000, Opal 570, 1/100, RRID:AB_2924310
- AR, ab108341, Recombinant Anti-Androgen Receptor antibody EPR179(2), Monoclonal, 1036164-3, Rabbit, 1/1000, Opal 690, 1/100, RRID:AB_10865716
- PanCK ab217916 Anti-pan Cytokeratin antibody (ab217916) polyclonal polyclonal GR3409120-4 Rabbit 1/400 Opal 780/ TSA-DIG 1/15 1/50, n/a
- Anti-Chromogranin A antibody (ab45179) polyclonal, polyclonal, GR3417483-2, rabbit, 1/800 Opal 620 1/100 RRID:AB_726879

Secondary antibodies used for mIHC were the anti-rabbit Akoya Rabbit HRP (cat ARR1001KT, Akoya Biosciences) and the anti-mouse Mouse Superboost (cat B40961, Thermo Fisher Scientific). The tyramide-conjugated fluorophores were Opal 480 (cat FP1500001KT, Akoya Biosciences; 1;75); Opal 520 (cat FP1487001KT, Akoya Biosciences; 1:75); Opal 570 (cat FP1488001KT, Akoya Biosciences; 1:100); Opal 690 (cat FP1497001KT, Akoya Biosciences; 1:100); Opal 780 (cat FP1501001KT, Akoya Biosciences, Opal 780 dilution 1:15, TSA-DIG dilution 1:50).

for single IHC:
- cMyc, ab32072, Recombinant Anti-c-Myc antibody [Y69] - ChIP Grade (ab32072), Y69, Monoclonal, 1025399-7, Rabbit, 1/100, N/A, N/A, RRID:AB_731658
- Pten (Cell Signaling), 9188S, PTEN (D4.3) XP, Rabbit, mAb, D4.3, Monoclonal, 6, Rabbit, 1/125 N/A N/A, RRID:AB_2253290
- pAKt(Cell Signaling), 4060S, P-AKt (S473) (D9E) XP(R), Rabbi,t mAb D9E Monoclonal, 25, Rabbit 1/100 N/A N/A,RID:AB_2315049
- nMYC, 51705S (Cell Signaling), N-Myc (D4B2Y), D4B2Y, Monoclonal, n/a, Rabbit, 1/100, N/A N/A, RRID:AB_2799400
- ERG, ab92513, Anti-ERG antibody [EPR3864], EPR3864, Monoclonal, 30, Rabbit, 1/1000, N/A, N/A, RRID:AB_2630401

The secondary antibody used in IHC was the Poly-HRP IgG reagent from the BOND Polymer Refine Detection Kit (cat DS9800, Leica Biosystems).

Validation

We followed the strategy to ensure a stringent selection of primary antibodies and conditions. We tested antibodies that recognize a single protein species on immunoblot and that are blocked by an excess of the protein that was used to generate them, as shown on the vendor's web site or data sheet. Each antibody staining was optimized using appropriate tissue (frozen and formalin fixed paraffin embedded), with the aim of obtaining the best signal-to-noise ratio. For each antibody we tested a range of different concentrations and epitope retrieval with citrate buffer at pH 6.0 for 20 minutes. The controls used for the optimization included tissue expressing the target antigen and tissue devoid of the target protein, and we included a further negative control omitting the primary antibody. Our main validation purpose was to confirm that the antibody staining was matching the expected positive and negative controls on the target tissues and locating in the right subcellular compartment. To ensure the right results on human tissue, we compared our pattern of staining with the results generated from the Human Protein Atlas (Uhlén M et al., Tissue-based map of the human proteome. Science (2015) PubMed: 25613900 DOI: 10.1126/science.1260419).

Eukaryotic cell lines

Policy information about cell lines and Sex and Gender in Research

Cell line source(s)

1) human: 22Rv1-N-Myc-Rb1KO (PMID: 31260412; PMID: 34099734) originating from ATCC 22Rv1 cat#CRL-2505™ (a human, male, prostate carcinoma epithelial cell line derived from a xenograft that was serially propagated in mice)
2) mouse primary Normal Associated Fibroblasts (NAFs) derived from a prostate 3 month old males of a pure background of c57/Bl6

3) mouse primary Normal Associated Fibroblasts (NAFs) derived from a prostate 6 month old males of a pure background of FVBN
4) mouse primary epithelial cells derived from a prostate 6 month old males of T-ERG
5) mouse primary epithelial cells derived from a prostate 8 weeks old males of PRN

Authentication

human cells were authenticated by ATCC using short tandem repeated profiling, mouse primary NAF derived from the pure C57/Bl6 genetic background male, that has been back crossed to maintain its background, mouse primary NAF derived from the pure FVBN genetic background male, that has been back crossed to maintain its background

Mycoplasma contamination

human and murine cells were tested routinely for mycoplasma contamination. All cell lines tested negative for mycoplasma. Human cells were not used at passages higher than 50.

Commonly misidentified lines
(See ICLAC register)

no commonly misidentified cell lines were used in the study

Animals and other research organisms

Policy information about studies involving animals; ARRIVE guidelines recommended for reporting animal research, and Sex and Gender in Research

Laboratory animals

In this study, only males were utilized. All animals used in this study received humane care in compliance with the principles stated in the Guide for the Care and Use of Laboratory Animals (National Research Council, 2011 edition), and the protocol was approved by the Institutional Animal Care and Use Committee of Weill Cornell Medicine, Dana-Farber Cancer Institute and Columbia University Irving Medical Center.

Mice are detailed in Table S1 which includes the following information: Mouse model, Designation, Source of reference, Age/ time-point of harvesting tissue and, Number of mice that have been used in studies:
- Tmprss2tm1.1(ERG)Sho, T-ERG (MT), RRID:MGI:5578645; PMID: 23512661, 6 months, 5 mice

- Nkx3.1creERT2;Ptenf/f ; EYFPf/+ ,NP (MT), PMID: 22815528, PMID: 28411207 (Cory Abate-Shen lab); RRID:IMSR_JAX:033751, 8 months, 3 mice

- Tg(ARR2/Pbsn-MYC)7Key, Hi-MYC (MT), P RRID:MGI:5486199; PMID: 14522256, 6 months, 5 mice

-Pb-Cre4 +/-;Pten f/f; LSL-MYCN +/+; Rb1 f/f, PRN (MT), PMID: 27728805, PMID: 34099734 (David Rickman Lab), 8 weeks, 3 mice

- FVB/N- pure background, WT for T-ERG and Hi-Myc , FVB/N FVB/N (Stock #: 207)_Charles River Laboratories (CRL); RRID:IMSR_CRL:207, 6 months, 5 mice

- B6129SF2/J - littermates, WT for NP, PMID: 22815528, PMID: 28411207 (Cory Abate-Shen lab), 8 months, 3mice

- C57BL6/129x1/SvJ) - littermates ,WT for PRN, PMID: 27728805, PMID: 34099734 (David Rickman Lab), 8 weeks, 5 mice

- C57Bl/6Jn- pure background, B6 (additional WT) , C57BL/6J (Stock #: 000664)_The Jackson Laboratory; RRID:IMSR_JAX:000664, 6 months, 5 mice

- B6129SF2/J- pure background, B6.129 (additional WT), B6129SF2/J (Stock #: 101045)_The Jackson Laboratory; RRID:IMSR_JAX:101045, 6 months, 4 mice

In addition, we used histology samples of mice provided by Dr. Goodrich; PBCre4;Ptenf/f;Rb1f/f (DKO) and PBcre4;Ptenf/f;Rb1f/f;Trp53f/f (TKO) models. All information bout these mice were reported in PMID: 28059767

Mice were housed in Research Animal Resource Center (RARC). RARC utilizes “shoe-box” cages (Thoren Caging Systems, Hazelton, PA) for housing mice ranging from 63.0 in2 to 75 in2 of floor space. Cages are changed weekly, with the wire bar lid and filter top replaced every other week. Feed (LabDiet 5053, PMI, St. Louis, MO) is gamma irradiated and replenished as needed during cage change. Bedding and enrichment are ‘bulk’2 sterilized and water is reverse osmosis (RO)-filtered3 and acidified. Cage components are washed at a high temperature but are not autoclaved.

Cage change is conducted within an animal transfer station (ATS) or a biological safety cabinet (BSC) using broad-spectrum disinfectants for hand and forceps disinfection.HEPA-filtered room air is supplied to each cage and the rack effluent is exhausted directly into the building’s exhaust system.

Cages are changed weekly in either a HEPA-filtered vertical flow change station or a Class 2 Type A biological safety cabinet3. The animal holding room is maintained at 72 ± 2 °F (21.5 ± 1 °C), relative humidity between 30% and 70%, and a 12:12 hour4 light:dark photoperiod.

Mice are tested for pathogens using MFIA and PCR methods. Testing laboratory is IDEXX/CRL and testing frequency depending on the pathogen is performed bimonthly, semiannually and annualy.

Wild animals	No wild animals were used in the study.
Reporting on sex	All animals used in our study were males.
Field-collected samples	No field collected samples were used in the study.
Ethics oversight	All animals used in this study received humane care in compliance with the principles stated in the Guide for the Care and Use of Laboratory Animals (National Research Council, 2011 edition), and the protocol was approved by the Institutional Animal Care and Use Committee of Weill Cornell Medicine, Dana-Farber Cancer Institute and Columbia University Irving Medical Center.

Note that full information on the approval of the study protocol must also be provided in the manuscript.

Flow Cytometry

Plots

Confirm that:

- [x] The axis labels state the marker and fluorochrome used (e.g. CD4-FITC).
- [x] The axis scales are clearly visible. Include numbers along axes only for bottom left plot of group (a 'group' is an analysis of identical markers).
- [x] All plots are contour plots with outliers or pseudocolor plots.
- [x] A numerical value for number of cells or percentage (with statistics) is provided.

Methodology

Sample preparation	Dissociated murine prostate cells were prepared as described previously (Drost J, et al. Organoid culture systems for prostate epithelial and cancer tissue. Nat Protoc 11, 347-358 (2016). Briefly, mouse prostate tissues were digested in Advanced DMEM/F12/Collagenase II (1.5mg/ml)/Hyaluronidase VIII (1000 u/ml) (Thermo Fisher Scientific) plus 10 µM Y-27632 (Tocris) for 1 hour at 37°C with 1500 rpm mixing, continuously agitated. Subsequently, after centrifuging at 150 g for 5 min at 4°C, digested cells were suspended in 1 ml TrypLE with 10 µM Y-27632 and digested for 15 min at 37°C and neutralized in aDMEM/F12/FBS (0.05%). Dissociated cells were subsequently passed through 70 µm and 40 µm cell strainers (BD Biosciences, San Jose, CA) to obtain a single cells suspension.<br><br>FACS sorting: Samples were resuspended in 1x PBS and sorted by Flow Cytometry (Becton-Dickinson Aria II and/or Becton-Dickinson Influx) for 4',6-diamidino-2-phenylindole (DAPI) to enrich for living cells.<br><br>Similarly, human prostate tissues were first digested in aDMEM/F12/Collagenase II (1.5mg/ml)/Hyaluronidase VIII (1000 u/ml; Thermo Fisher Scientific) plus 10 µM Y-27632 (Tocris) for 1 hour at 37°C with 1500 rpm mixing, continuously agitated. Subsequently, after centrifuging at 150 g for 5 min at 4°C, digested cells were suspended in 1 ml TrypLE with 10 µM Y-27632 and digested for 15 min at 37°C and neutralized in aDMEM/F12/FBS (0.05%).<br><br>FACS sorting: Dissociated cells were subsequently passed through 70 µm and 40 µm cell strainers (BD Biosciences, San Jose, CA) to get single cells. Samples were resuspended in 1x PBS and sorted for DAPI to enrich living cells.
Instrument	BD FACS Aria II instrument (BD Biosciences, San Jose, CA), Becton-Dickinson Influx
Software	FlowJo software (FlowJo 10.8.1, Treestar, San Carlos, CA).
Cell population abundance	For all samples that were cell sorted for single-cell profiling by 10X, live cells that were DAPI negative were 40-70% of total cells
Gating strategy	Cells were selected first based on FSC-A and SCC-A followed by singlet selection for both FSC and SSC. Then live cells were selected based on DAPI staining (DAPI negative) cells which were sorted for 10x experiments (DAPIneg only)

- [x] Tick this box to confirm that a figure exemplifying the gating strategy is provided in the Supplementary Information.
